# Preconditioning strategies to prevent acute kidney injury

**DOI:** 10.12688/f1000research.21406.1

**Published:** 2020-04-03

**Authors:** Martin Richard Späth, Felix Carlo Koehler, Karla Johanna Ruth Hoyer-Allo, Franziska Grundmann, Volker Burst, Roman-Ulrich Müller

**Affiliations:** 1 Department II of Internal Medicine and Center for Molecular Medicine Cologne, University of Cologne, Faculty of Medicine and University Hospital Cologne, Cologne, NRW, 50937, Germany; 2 Cologne Excellence Cluster on Cellular Stress Responses in Aging-Associated Diseases, University of Cologne, Cologne, NRW, 50931, Germany

**Keywords:** acute kidney injury, AKI, preconditioning, hypoxia, hypoxic preconditioning, RIPC, ischemic preconditioning, diet, dietary restriction, caloric restriction, protein restriction, fasting, contrast induced nephropathy, cardiopulmonary bypass

## Abstract

Acute kidney injury is a common clinical disorder resulting in significantly increased morbidity and mortality. However, despite extensive research, strategies for prevention or treatment are still lacking in routine clinical practice.

Already decades ago, several preconditioning strategies (e. g. ischemic/hypoxic preconditioning and calorie restriction) have been published and their extraordinary effectiveness - especially in rodents - has raised the hope for powerful clinical tools to prevent acute kidney injury. However, the underlying mechanisms are still not completely understood and translation to the clinics has not been successful yet. In this review, the most attractive strategies and the current mechanistic concepts are introduced and discussed. Furthermore, we present clinical trials evaluating the feasibility of preconditioning in the clinical setting.

## Introduction

Acute kidney injury (AKI) is a highly relevant problem in clinical practice and is associated with an increased risk of mortality ^1, 2^, the development of chronic kidney disease, and cardiovascular events ^3, 4^. The incidence of AKI has increased in recent years, and, in the context of the demographic change, it is likely that a plateau has not been reached yet ^5, 6^.

Imbalances between circulatory demands and perfusion leading to renal ischemia as well as toxic insults are frequent causes of AKI and are often observed as adverse events of medical interventions (for example, major surgery or chemotherapy). In both cases, loss of cell adhesion molecules, cell polarity, and death of tubular epithelial cells lead to cell detachment with subsequent urinary tract dysfunction ^7^. Intensive monitoring and adequate fluid management at the time of diagnosis constitute the standard therapy. However, an effective treatment for established AKI is still missing. Apart from maintaining euvolemia, atraumatic/minimally invasive surgical techniques, and avoidance of potentially nephrotoxic substances, no preventive measures have been proven to exert a protective benefit in clinical practice ^8^.

Animal models have been established for both types of AKI primarily using rodents and employing ischemia-reperfusion injury (IRI) by transient clamping of the renal vessels ^9– 11^ or toxic tubular injury induced by the intraperitoneal injection of cisplatin ^12– 14^. In both models, various preconditioning algorithms have proven to be very effective. In general, the term “preconditioning” describes the strategy of activating the body’s own stress resistance mechanisms, thereby mitigating consecutive harm ^15^, and commonly exploits the concept of hormesis.

In this review article, we discuss known and effective strategies of preconditioning. Considering promising results from animal models and first results in clinical trials, we will focus on strategies targeting either the cellular response to hypoxia or nutrient availability: ischemic preconditioning (IPC) ^16^, remote IPC (RIPC) ^17^, hypoxic preconditioning (HP) ^18^, prolyl-hydroxylase (PHD) inhibition (PHDi) ^19^, and dietary interventions ^9^.

## Ischemic preconditioning

The oldest known procedure is IPC: repetitive periods of short-term sublethal organ ischemia enhance the resistance against cellular stress and mitigate the damage of subsequent profound ischemic injury ^16^. The underlying mechanisms have not been fully deciphered yet. So far, it has been assumed that the repetitive stimuli lead to a release of various chemical messengers (for example, calcium ions, reactive oxygen species, and hydrogen sulfide), vasoactive substances (for example, adenosine, bradykinin, opioids, and urocortins), neurotransmitters and hormones (for example, acetylcholine and angiotensin) as well as cytokines (for example, tumor necrosis factor-alpha [TNF-α], interleukin-6, and prostaglandins) activating G protein–coupled receptors ^20, 21^. A number of pathways have been implicated to be involved downstream of these mediators. First is the activation of nitric oxide (NO) and NO synthase (NOS) by activation of protein kinase C (PKC), phosphoinoside 3 kinase (PI3K/Akt), and the guanylatcyclase leading to the activation of ATP-dependent potassium channels (K _ATP_) and the priming of mitochondrial permeability transition pore (MPTP) ^20, 22– 26^. Second is the reperfusion injury salvage kinase (RISK) pathway by activation of PI3K/Akt, 70 ribosomal protein S6 kinase (p70S6K), glycogen synthase kinase 3b (GSK3b), and the inhibition of MPTP opening ^20, 21, 26– 29^. Third, the survival activating factor enhancement (SAFE) pathway gets activated by activation of glycoprotein 130 (gp130) or TNF-α receptors, the Janus-activated kinase (JAK) signal transducer, and the stimulation of the activator of transcription (STAT) ^20, 21, 26, 29– 31^. Furthermore, a role for SIRT1-mediated NAD ^+^-dependent deacetylation ^32^ and mitochondrial biogenesis ^33^ has recently been shown.

## Remote ischemic preconditioning

Although IPC shows promising results in animal models and clinical pilot trials, it is obvious that translation to the clinical setting is restricted to surgical settings and therefore is not easily feasible. Consequently, aiming to exploit similar mechanisms, the strategy of RIPC was developed. Through RIPC, an organ (for example, the heart) can be protected from damage by applying repetitive short-term sublethal periods of ischemia to a remote tissue (for example, the kidney) ^34^. This protection goes along with a gene regulatory effect in the target organ ^35^. Several different mediators of this distant effect have been hypothesized in the past: on the one hand, a neuronal effect in which the afferent neurons of the preconditioned organ provide protection of the remote organ has been described ^36^. On the other hand, a humoral effect by various mediators has been described in several publications and the actual key candidates remain elusive. More recently, a very elegant study using the ischemia-reperfusion model of the heart could show that activation of hypoxia signaling in skeletal muscles increases the production of alpha-ketoglutarate (alphaKG). Circulating alphaKG induces kynurenic acid (KYNA) formation in the liver, and KYNA itself was sufficient to protect the heart from ischemic injury ^37^. Furthermore, a humoral activation of various cellular pathways (for example, by NO ^38^, connexin 43 ^39^, or hypoxia-inducible factor 1 alpha [HIF-1α] ^40^) has been published. The major advantage of RIPC lies in its simple clinical applicability since the remote effect can also be achieved by repetitive inflation of a blood pressure cuff on the arm or thigh. In a randomized, double-blind, sham-controlled clinical trial investigating the effect of RIPC prior to elective coronary angiography, a significant reduction of the incidence of contrast media–induced acute injury (defined as an increment of serum creatinine of at least 0.5 mg/dL or a relative increase of more than 25% over baseline value within 48 hours) could be shown for the intervention group in a cohort of 100 patients with chronic kidney disease (that is, serum creatinine of more than 1.4 mg/dL or estimated glomerular filtration rate [eGFR] of less than 60 mL/min * 1.73 m ^2^) and at high risk of developing contrast medium–induced kidney injury according to the Mehran risk score ^41^. In another randomized, prospective, multicenter, and double-blind clinical trial investigating 222 patients with a reduced eGFR (that is, <40 mL/min per 1.73 m ^2^ or between 40 and 60 mL/min per 1.73 m ^2^) and two or more risk factors (age ≥75 years, diabetes mellitus, or heart failure New York Heart Association [NYHA] III or IV) undergoing elective coronary angiography or percutaneous transluminal coronary angioplasty (or both), no significant difference for any study group could be shown regarding the incidence of contrast media–induced nephropathy (primary endpoint), change of serum creatinine, or change of eGFR (secondary endpoints) ^42^. The comparability of both studies is limited because the population of the latter trial included fewer patients with diabetes mellitus (~50 to ~62%, respectively) and the Mehran score differed markedly (~8 to 13). Besides, the incidence of contrast media–induced nephropathy in the control group of the trial by Roubille *et al*. ^42^ was reduced in comparison with their own database (4.5 to 28%) and in comparison with the trial by Er *et al*. ^41^ (20%). Furthermore, the volume of contrast media used was much smaller in the trial by Roubille *et al*. ^42^ (~75 mL) in comparison with the trial by Er *et al*. ^41^ (~120 mL).

In addition, the clinical relevance of contrast-induced nephropathy (CIN) has been debated extensively in recent years, supporting the idea that its incidence is far overrated in clinical practice; this, in turn, limits the value of the used model ^43^. The largest trials examining the protective potential of RIPC were performed in cohorts undergoing surgery on cardiopulmonary bypass. Unfortunately, owing to major differences in both outcome and patient characteristics, these studies, in line with the data on CIN, have not provided conclusive results. Zarbock *et al*. conducted a prospective randomized, double-blind, and sham-controlled multicenter trial enrolling 240 patients who underwent cardiac surgery with the use of cardiopulmonary bypass ^44^. RIPC led to a lower incidence of AKI (primary endpoint), a reduced need for renal replacement therapy (RRT), and a shorter length of stay in the intensive care unit (ICU). These results were highly promising at first; however, two much larger randomized, double-blind trials published in 2015 dampened the enthusiasm. The RIPHEART trial examined RIPC in 1403 subjects undergoing coronary artery bypass graft (CABG) surgery and did not detect any benefit concerning the primary endpoint (death, myocardial infarction, stroke, AKI, or length of stay in the ICU) ^45^. The same holds true for the ERICCA trial ^46^. Here, RIPC did not result in improved resistance to cardiovascular and cerebrovascular events within 12 months after surgery (primary endpoint) and perioperative myocardial infarction, acute myocardial infarction, AKI, length of stay on the ICU, ejection fraction, and quality of life (secondary endpoints) in 1612 patients after CABG with or without cardiac valve surgery (801 RIPC and 811 sham). An important aspect that has been discussed to explain some of the differences between these trials is the different mode of anesthesia that may interfere with the potential of RIPC. In ERICCA, the anesthetic procedure was not standardized ^46^ whereas it was limited to inhalation anesthesia in Zarbock *et al*. and intravenous anesthesia using propofol in RIPHEART. Furthermore, Zarbock *et al*. explicitly enrolled only individuals at a high risk of AKI defined by a Cleveland Clinic Foundation score of 6 or higher. In RIPHEART, there was no stratification for the risk of AKI and the participants showed a moderate risk for death 30 days after surgery (mean Logistic EuroSCORE ^47, 48^ for both groups: 4.2). In ERICCA, patients were eligible only with a EuroSCORE of 5 or higher without any specific risk assessment for AKI. In a meta-analysis of RIPC in the prevention of AKI in patients undergoing CABG surgery, a benefit of this pre-treatment could be demonstrated only in the subgroup that received inhalation anesthesia. Here, it is important to note that volatile anesthetics themselves are being discussed as preconditioning agents ^49, 50^. Nonetheless, RIPC appeared to have a potential additive effect to this protective potential in this study. With regard to the development of dialysis-dependent kidney injury, there was no advantage in any of the subgroups examined ^51^. From the perspective of the authors, owing to the different risk stratification in the selection of subjects and non-uniform operating procedures, a final assessment regarding the potential of RIPC in the clinical setting is currently not possible.

## Hypoxic preconditioning and prolyl-hydroxylase inhibition

Although for a couple of years the field focused on driving RIPC toward a potential clinical use, other modes of preconditioning have regained increasing attention more recently. In 1994, a model for HP was published ^52^. Six-day-old rats that were preconditioned by subjecting them to an ambient oxygen content of 8% for 3 hours showed significantly reduced cerebral infarct zones following unilateral occlusion of the carotid artery ^52^. Altered HIF signaling could be identified as a mediator of this effect ^18^. Even though HP, like IPC, cannot be directly transferred to the clinical setting, activation of HIF can be induced pharmacologically, allowing potential future interventions in the patient setting. HIFs are heterodimeric proteins consisting of a hypoxia-regulated HIF-α and a constitutive HIF-β subunit ^53^. Under normoxia, PHDs hydroxylate specific proline residues of the HIF-α subunit, inducing its proteasomal degradation. Hypoxia inhibits PHDs and allows the nuclear accumulation of HIF-α where the assembly of dimers of α- and β-subunit leads to the transcription of numerous target genes that have been hypothesized to be involved in the protective effect (for example, erythropoietin, vascular endothelial growth factor [VEGF], and heme oxygenase-1) ^54, 55^. PHD inhibitors are available and have been shown to mediate a protective effect regarding renal IRI comparable to HP using a rat model ^19^. Interestingly, in the meantime, a PHD inhibitor—roxadustat—has been approved for the treatment of anemia in patients with dialysis-dependent and non-dialysis-dependent chronic kidney disease in China ^56– 58^ and Japan. More data on roxadustat were recently presented at Kidney Week ^59– 61^ aiming at approval for the US and Europe. Additionally, various other PHD-inhibiting compounds are being tested in clinical trials ^53^. A double-blind, randomized, single-center phase II trial (ClinicalTrials.gov Identifier: NCT01920594) investigating a possible reduction in neurological, renal, or cardiac ischemia (or a combination of these) by PHDi prior to elective aortic aneurysm surgery has been completed but not fully published yet.

## Caloric restriction

Apart from targeting the cellular response to hypoxia, dietary interventions have been shown to be one of the most promising strategies in organoprotection. Calorie restriction (CR) has long been known to mediate life-span extension, a finding that was first demonstrated in a rat model in 1935 ^62^ and confirmed in various different species, including primates ^63^. Importantly, as known for many longevity-inducing interventions, CR leads to a profound and conserved increase in organismal and cellular stress resistance ^64, 65^. More recent work could show that changes in nucleolar biology are a shared mechanism of several life span–extending interventions with CR inducing a significant reduction in nucleolar size in *Caenorhabditis elegans*, fruit flies, mice, and humans ^66^. Importantly, nucleolar size early in life was also predictive of life span in the nematode model. Regarding the kidney, Mitchell *et al*. could show that a short-term reduction in food intake (to 70%) protected against murine renal IRI ^9^. Improved insulin sensitivity and reduced insulin/IGF-1 signaling and increased expression of antioxidant defense enzymes were hypothesized to be among the key mechanisms for protection against renal and hepatic IRI as well as genotoxic or chemotherapeutic stress ^9, 67– 69^. Recently, it was shown that the CR-mediated improvement of insulin sensitivity is mediated by mTORC2 signaling. However, disruption of mTORC2 signaling inducing insulin resistance in a *Rictor* knockout mouse model did not diminish the increase of fitness and life span ^70^, indicating a potential mechanistic difference in longevity and stress resistance. Although the CR-mediated protective effect has been published in several mammals, effectiveness in humans has not yet been clearly demonstrated ^71, 72^. Yet there is first evidence of feasibility and potential efficacy in humans. The safety of a preoperative calorie- and protein-restricted diet in healthy kidney donors and obese patients undergoing bariatric surgery was shown by Jongbloed *et al*. ^73^. Furthermore, feasibility was addressed in living kidney donors ^74^. A large-scale trial investigating permissive underfeeding compared with standard enteral feeding in critically ill patients demonstrated a significantly lower rate of RRT in the group with a calorie-restricted enteral feeding protocol ^71, 72^. Recently, we studied the effects of a 7-day preoperative CR on renal function in a randomized controlled clinical trial ^75^. Eighty-two patients at risk for post-surgery AKI were randomly assigned 1:1 to receive either a formula diet containing 60% of their daily energy requirement or *ad libitum* food for 7 days prior to elective cardiac surgery involving cardiopulmonary bypass. Although CR had no impact on the primary endpoint (the increase of serum creatinine at 24 hours after cardiac surgery), there was a significant between-group difference with a favorable effect of CR on creatinine kinetics at 48 hours and at discharge. Additional subgroup analyses suggested that the positive effect appeared to be most prominent in men and obese individuals with a body mass index of more than 25 kg/m ^2^ ^75^. In parallel, a second randomized controlled clinical trial for preventing AKI in patients undergoing percutaneous coronary intervention was performed to determine the feasibility and effectiveness of pre-interventional CR. As in the above-mentioned trial, patients were randomly assigned either to receive a formula diet containing 60% of their calculated daily energy expenditure or to *ad libitum* food intake. Again, beneficial effects were detected only in post-hoc subgroup analyses ^76^. Although the findings of these clinical trials did not reflect the effects of CR seen in animal experiments, the studies could show that the intervention is safe and feasible even in a morbid patient population. The fact that the magnitude of the observed effects is smaller than in the rodent models may be due to several aspects. It is still unknown how long a diet must be applied in humans, how much the caloric content has to be restricted, and how the most potent dietary regimen should be designed ^75^. Given these caveats of implementing CR in the clinical setting, it is extremely important to have a better understanding of both the molecular mechanisms underlying CR-mediated organoprotection and the ideal dietary interventions to obtain these effects. From the authors’ point of view, this is necessary in order to further develop targeted approaches (for example, by drugs or targeted dietary interventions) for improving feasibility and effect size in a clinical setting.

## Protein restriction

There is ample evidence that CR does not mediate stress resistance through mere reduction of calories, and several other dietary interventions that modulate specific dietary components have shown beneficial effects in organ injury. Here, protein restriction (PR) is an important example, and PR has been demonstrated to confer additive effects to CR ^77^. Interestingly, restriction of any single essential amino acid appears to be sufficient to mediate systemic adaptive responses leading to metabolic benefits ^78– 80^. Regarding renal organ protection, an important aspect was highlighted by a study published in 2015 showing that PR (with identical calorie intake in both groups) prior to hepatic IRI caused strong protective effects similar to those of CR and that these positive effects could be reversed by the addition of sulfur-containing amino acids ^81, 82^. Mechanistically, restriction of sulfur-containing amino acids caused an increase of hydrogen sulfide (H _2_S) formation by activation of the transsulfuration pathway ^81^ and addition of H _2_S induced cellular stress resistance ^83– 85^. Hence, both H _2_S donors (for example, MESNA ^86^) and diets reduced in sulfur-containing amino acid intake ^87^ may be future strategies to transfer the potential of CR to the patient setting. A clinical trial (ClinicalTrials.gov Identifier: NCT03715868) investigating a non-dairy (significantly reduced in sulfur amino acids) formula diet prior to cardiac surgery was recently initiated at our center.

## Fasting-mimicking and ketogenic diets

Intermittent or periodic fasting enables the activation of cellular signal transduction similar to that of CR with preserved nourishment ^88^. Fasting-mimicking diets (FMDs) are a tool to reach comparable effects, and their safety and feasibility have been proven in several phase I and phase II studies ^89– 91^. Mechanistically, FMD results in cellular and metabolic effects similar to those of CR, including improved glucose homeostasis and insulin sensitivity, as well as improved cellular stress adaptation ^89, 92^ (for example, by modulation of the mechanistic target of rapamycin [mTOR] pathway ^93^). It will be interesting to see whether these approaches have a role in organoprotection as well.

mTOR is an evolutionary conserved protein kinase orchestrating growth and metabolism. By inducing autophagy and by the reduction of protein translation leading to decreased proteotoxic and oxidative stress, mTOR inhibition results in cellular stress resistance ^94, 95^. mTOR is regulated through nourishment, and dietary inhibition of the mTOR pathway can be achieved either by restriction of the branched chain amino acids (BCAAs) valine, leucine, and isoleucine or by changes in the ratio of macronutrients replacing proteins with carbohydrates ^96– 98^. Increased exposure to BCAA is associated with hyperphagia, obesity, insulin resistance, and mortality ^99– 101^. Additionally, a protein-to-carbohydrate ratio of 0.07 resulting in low-protein and high-carbohydrate diet leads to improved stress resistance, health, and life span ^82, 98, 102^. Strikingly, low-sugar diets also revealed beneficial effects on health and life span in *C. elegans* ^103^. On the other hand, glucose supplementation did not interfere with fasting-induced renal protection in the ischemia-reperfusion mouse model ^104^. Because the optimal diet for humans in the context of organ protection remains unknown, there is much room for improvement. Here, clarification whether reduction of specific amino acids or the changes in ratio of the macronutrients are the key drivers to improve metabolism, fitness and health in humans will be required.

Ketogenic diets are high in fat and very low in carbohydrates and result in synthesis of ketone bodies and exceeding β-oxidation of fatty acids. Similar to CR, ketogenic diets extend the life span in rodents with preserved physiological functions but do not lead to malnourishment ^105^. Ketone bodies, such as β hydroxybutyrate, suppress oxidative stress, resulting in nephroprotection ^106^. Owing to their additional neuroprotective effects, ketogenic diets have been proven to be feasible and safe in medical use in human pharmacoresistant epilepsy ^107^. Given that CR in rodents actually does induce ketogenesis because of the nature of the feeding cycles ^108^, ketogenic diets may be another promising strategy to ameliorate AKI in a clinical setting.

## Summary

**Figure 1.**
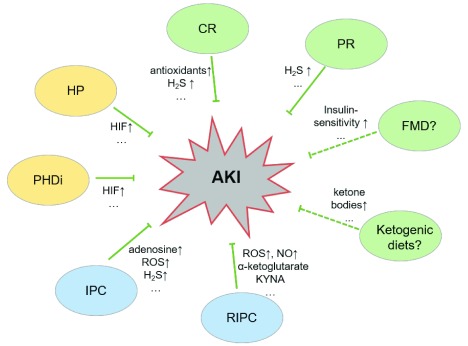
Schematic illustration of the mechanisms involved in various preconditioning strategies. The different modes of preconditioning have been shown, or are supposed, to alleviate acute kidney injury by production of chemokines or metabolites. AKI, acute kidney injury; CR, calorie restriction; FMD, fasting-mimicking diet; H2S, hydrogen sulfide; HIF, hypoxia-inducible factor; HP, hypoxic preconditioning; IPC, ischemic preconditioning; KYNA, kynurenic acid; PHDi, prolyl-hydroxlase inhibition; PR, protein restriction; RIPC, remote ischemic preconditioning; ROS, reactive oxygen species. Bar-headed lines indicate inhibition, dashed lines and question marks indicate hypotheses, “…” indicates that not all mediators are displayed, and ↑ indicates increase.

Taken together, recent experiments in animal models have helped to increase our understanding of preconditioning in AKI (see Figure 1), although clear-cut clinical effectiveness in humans has not been proven yet. Consequently, dietary interventions that have been tested to date in humans will probably not solve the problem. Nonetheless, given the tremendous effects in animal models in combination with the unmet clinical need, such research is of high importance. Direct pharmacological or optimized tailored dietary targeting of the molecular players may be the more straightforward approach in humans but will not be possible without detailed knowledge of the molecular mechanisms. Given that different modes of preconditioning are potentially based on similar mechanisms, comparative analyses may have a significant added value. In a recent study on HP and CR in the mouse model of cisplatin-induced kidney injury using an integrative analysis of transcriptomics, proteomics, and N-degradomics, we found that mRNA expression only moderately predicted protein expression. But the more the mRNA and the proteome dissociated, the higher was the serum creatinine in the individual animal. N-degradomic studies revealed extracellular, specific proteolytic complement activation that can be alleviated by these two preconditioning methods ^12^; this is in line with results of other groups showing that protease inhibition is a potential therapeutic approach ^109, 110^. Further studies comparing two different modes of preconditioning (HP-CR and HP-PHDi) in murine renal IRI models are being carried out, revealing shared mechanisms reflected by overlapping pathways and common regulation of target genes in association with the clinical outcome by integrative multi-omics approaches ^111, 112^. Numerous other basic scientific and clinical studies on these procedures are under way. This improved knowledge of the molecular mechanisms involved will be crucial to translate future protective strategies into the clinical setting (for example, using targeted pharmacological approaches).

## Abbreviations

alphaKG, α-ketoglutarate; AKI, acute kidney injury; BCAA, branched chain amino acid; CABG, coronary artery bypass graft; CIN, contrast-induced nephropathy; CR, calorie restriction; eGFR, estimated glomerular filtration rate; FMD, fasting-mimicking diet; H _2_S, hydrogen sulfide; HIF, hypoxia-inducible factor; HP, hypoxic preconditioning; ICU, intensive care unit; IPC, ischemic preconditioning, IRI, ischemia-reperfusion injury; KYNA, kynurenic acid; MPTP, mitochondrial permeability transition pore; mTOR, mechanistic target of rapamycin; NO, nitric oxide; PHD, prolyl-hydroxylase; PHDi, prolyl-hydroxylase inhibition; PI3K, phosphoinoside 3 kinase; PR, protein restriction; RIPC, remote ischemic preconditioning; RRT, renal replacement therapy; TNF-α, tumor necrosis factor-alpha

## References

[1] Oppert M Engel C Brunkhorst FM: Acute renal failure in patients with severe sepsis and septic shock--a significant independent risk factor for mortality: Results from the German Prevalence Study. *Nephrol Dial Transplant.* 2008;23(3):904–9. 10.1093/ndt/gfm610 18065435

[2] Susantitaphong P Cruz DN Cerda J: World Incidence of AKI: A Meta-Analysis. *Clin J Am Soc Nephrol.* 2013;8(9):1482–93. 10.2215/CJN.00710113 23744003 PMC3805065

[3] Leung KCW Tonelli M James MT: Chronic kidney disease following acute kidney injury-risk and outcomes. *Nat Rev Nephrol.* 2013;9(2):77–85. 10.1038/nrneph.2012.280 23247572

[4] Lim CC Tan CS Chia CM: Long-Term Risk of Progressive Chronic Kidney Disease in Patients with Severe Acute Kidney Injury Requiring Dialysis after Coronary Artery Bypass Surgery. *Cardiorenal Med.* 2015;5(3):157–63. 10.1159/000381068 26195967 PMC4478312

[5] Hsu Cy McCulloch CE Fan D: Community-based incidence of acute renal failure. *Kidney Int.* 2007;72(2):208–12. 10.1038/sj.ki.5002297 17507907 PMC2673495

[6] Baba M Shimbo T Horio M: Longitudinal Study of the Decline in Renal Function in Healthy Subjects. *PLoS One.* 2015;10(6):e0129036. 10.1371/journal.pone.0129036 26061083 PMC4464887

[7] Basile DP Anderson MD Sutton TA: Pathophysiology of acute kidney injury. *Compr Physiol.* 2012;2(2):1303–53. 10.1002/cphy.c110041 23798302 PMC3919808

[8] O’Kane D Baldwin GS Bolton DM: Preconditioning against renal ischaemia reperfusion injury: The failure to translate to the clinic. *J Nephrol.* 2019;32(4):539–47. 10.1007/s40620-019-00582-6 30635875

[9] Mitchell JR Verweij M Brand K: Short-term dietary restriction and fasting precondition against ischemia reperfusion injury in mice. *Aging Cell.* 2010;9(1):40–53. 10.1111/j.1474-9726.2009.00532.x 19878145 PMC3412229

[10] Johnsen M Späth MR Denzel MS: Oral Supplementation of Glucosamine Fails to Alleviate Acute Kidney Injury in Renal Ischemia-Reperfusion Damage. *PLoS One.* 2016;11(8):e0161315. 10.1371/journal.pone.0161315 27557097 PMC4996512

[11] Owji SM Nikeghbal E Moosavi SM: Comparison of ischaemia-reperfusion-induced acute kidney injury by clamping renal arteries, veins or pedicles in anaesthetized rats. *Exp Physiol.* 2018;103(10):1390–1402. 10.1113/EP087140 30091805

[12] Späth MR Bartram MP Palacio-Escat N: The proteome microenvironment determines the protective effect of preconditioning in cisplatin-induced acute kidney injury. *Kidney Int.* 2019;95(2):333–349. 10.1016/j.kint.2018.08.037 30522767

[13] Yang Y Yu X Zhang Y: Hypoxia-inducible factor prolyl hydroxylase inhibitor roxadustat (FG-4592) protects against cisplatin-induced acute kidney injury. *Clin Sci (Lond).* 2018;132(7):825–838. 10.1042/CS20171625 29581249

[14] Dugbartey GJ Bouma HR Lobb I: Hydrogen sulfide: A novel nephroprotectant against cisplatin-induced renal toxicity. *Nitric Oxide.* 2016;57:15–20. 10.1016/j.niox.2016.04.005 27095538

[15] Bein B Meybohm P: [Organ protection by conditioning]. *Anasthesiol Intensivmed Notfallmed Schmerzther*.2010;45(4):254–61; quiz 262. 10.1055/s-0030-1253094 20387183

[16] Murry CE Jennings RB Reimer KA: Preconditioning with ischemia: a delay of lethal cell injury in ischemic myocardium. *Circulation.* 1986;74(5):1124–36. 10.1161/01.cir.74.5.1124 3769170

[17] Przyklenk K Bauer B Ovize M: Regional ischemic 'preconditioning' protects remote virgin myocardium from subsequent sustained coronary occlusion. *Circulation.* 1993;87(3):893–9. 10.1161/01.cir.87.3.893 7680290

[18] Wang GL Jiang BH Rue EA: Hypoxia-inducible factor 1 is a basic-helix-loop-helix-PAS heterodimer regulated by cellular O2 tension. *Proc Natl Acad Sci U S A.* 1995;92(12):5510–4. 10.1073/pnas.92.12.5510 7539918 PMC41725

[19] Bernhardt WM Câmpean V Kany S: Preconditional activation of hypoxia-inducible factors ameliorates ischemic acute renal failure. *J Am Soc Nephrol.* 2006;17(7):1970–8. 10.1681/ASN.2005121302 16762988

[20] Stokfisz K Ledakowicz-Polak A Zagorski M: Ischaemic preconditioning - Current knowledge and potential future applications after 30 years of experience. *Adv Med Sci.* 2017;62(2):307–16. 10.1016/j.advms.2016.11.006 28511069

[21] Heusch G: Molecular basis of cardioprotection: signal transduction in ischemic pre-, post-, and remote conditioning. *Circ Res.* 2015;116(4):674–99. 10.1161/CIRCRESAHA.116.305348 25677517

[22] Weiss JN Korge P Honda HM: Role of the mitochondrial permeability transition in myocardial disease. *Circ Res.* 2003;93:292–301. 10.1161/01.RES.0000087542.26971.D4 12933700

[23] Downey JM Davis AM Cohen MV: Signaling pathways in ischemic preconditioning. *Heart Fail Rev.* 2007;12(3-4):181–8. 10.1007/s10741-007-9025-2 17516169

[24] Burley DS Hamid SA Baxter GF: Cardioprotective actions of peptide hormones in myocardial ischemia. *Heart Fail Rev.* 2007;12(3–4):279–91. 10.1007/s10741-007-9029-y 17516166

[25] Costa ADT Garlid KD: Intramitochondrial signaling: interactions among mitoKATP, PKCepsilon, ROS, and MPT. *Am J Physiol Heart Circ Physiol.* 2008;295(2):H874–82. 10.1152/ajpheart.01189.2007 18586884 PMC2519212

[26] Boengler K Schlüter K-D Schermuly RT: Cardioprotection in right heart failure. *Br J Pharmacol.* 2020. 10.1111/bph.14992 31995639 PMC7680005

[27] Hausenloy DJ Yellon DM: Reperfusion injury salvage kinase signalling: taking a RISK for cardioprotection. *Heart Fail Rev.* 2007;12(3–4):217–34. 10.1007/s10741-007-9026-1 17541822

[28] Przyklenk K Maynard M Darling CE: Aging mouse hearts are refractory to infarct size reduction with post-conditioning. *J Am Coll Cardiol.* 2008;51(14):1393–8. 10.1016/j.jacc.2007.11.070 18387442

[29] Annachhatre A Annachhatre S: Preconditioning in cardiac anesthesia…… where are we? *Ann Card Anaesth.* 2019;22(4):412. 10.4103/aca.ACA_116_18 31621678 PMC6813706

[30] Lecour S Suleman N Deuchar GA: Pharmacological preconditioning with tumor necrosis factor-α activates signal transducer and activator of transcription-3 at reperfusion without involving classic prosurvival kinases (Akt and extracellular signal-regulated kinase). *Circulation.* 2005;112(25):3911–8. 10.1161/CIRCULATIONAHA.105.581058 16344382

[31] Boengler K Hilfiker-Kleiner D Drexler H: The myocardial JAK/STAT pathway: from protection to failure. *Pharmacol Ther.* 2008;120(2):172–85. 10.1016/j.pharmthera.2008.08.002 18786563

[32] Nadtochiy SM Redman E Rahman I: Lysine deacetylation in ischaemic preconditioning: the role of SIRT1. *Cardiovasc Res.* 2011;89(3):643–9. 10.1093/cvr/cvq287 20823277 PMC3028968

[33] Basheer WA Fu Y Shimura D: Stress response protein GJA1-20k promotes mitochondrial biogenesis, metabolic quiescence, and cardioprotection against ischemia/reperfusion injury. *JCI Insight.* 2018;3(20). 10.1172/jci.insight.121900 30333316 PMC6237442

[34] Gho BCG Schoemaker RG van den Doel MA: Myocardial Protection by Brief Ischemia in Noncardiac Tissue. *Circulation.* 1996;94(9):2193–200. 10.1161/01.cir.94.9.2193 8901671

[35] Konstantinov IE Arab S Kharbanda RK: The remote ischemic preconditioning stimulus modifies inflammatory gene expression in humans. *Physiol Genomics.* 2004;19(1):143–50. 10.1152/physiolgenomics.00046.2004 15304621

[36] Ding YF Zhang MM He RR: Role of renal nerve in cardioprotection provided by renal ischemic preconditioning in anesthetized rabbits. *Sheng Li Xue Bao.* 2001;53(1):7–12. 11354802

[37] Olenchock BA Moslehi J Baik AH: EGLN1 Inhibition and Rerouting of α-Ketoglutarate Suffice for Remote Ischemic Protection. *Cell.* 2016;164(5):884–95. 10.1016/j.cell.2016.02.006 26919427 PMC4819986

[38] Küntscher MV Kastell T Altmann J: Acute remote ischemic preconditioning II: The role of nitric oxide. *Microsurgery.* 2002;22(6):227–31. 10.1002/micr.10042 12375287

[39] Brandenburger T Huhn R Galas A: Remote ischemic preconditioning preserves Connexin 43 phosphorylation in the rat heart *in vivo*. *J Transl Med.* 2014;12:228. 10.1186/s12967-014-0228-8 25159820 PMC4256705

[40] Albrecht M Zitta K Bein B: Remote ischemic preconditioning regulates HIF-1α levels, apoptosis and inflammation in heart tissue of cardiosurgical patients: A pilot experimental study. *Basic Res Cardiol.* 2013;108(1):314. 10.1007/s00395-012-0314-0 23203207

[41] Er F Nia AM Dopp H: Ischemic preconditioning for prevention of contrast medium-induced nephropathy: randomized pilot RenPro Trial (Renal Protection Trial). *Circulation.* 2012;126(3):296–303. 10.1161/CIRCULATIONAHA.112.096370 22735306

[42] Roubille F Macia J-C Ivanes F: Effects of remote ischemic conditioning on kidney injury in at-risk patients undergoing elective coronary angiography (PREPARE study): a multicenter, randomized clinical trial. *Sci Rep.* 2019;9(1):11985. 10.1038/s41598-019-47106-7 31427688 PMC6700075

[43] Wilhelm-Leen E Montez-Rath ME Chertow G: Estimating the Risk of Radiocontrast-Associated Nephropathy. *J Am Soc Nephrol.* 2017;28(2):653–9. 10.1681/ASN.2016010021 27688297 PMC5280012

[44] Zarbock A Schmidt C van Aken H: Effect of remote ischemic preconditioning on kidney injury among high-risk patients undergoing cardiac surgery: a randomized clinical trial. *JAMA.* 2015;313(21):2133–41. 10.1001/jama.2015.4189 26024502

[45] Meybohm P Bein B Brosteanu O: A Multicenter Trial of Remote Ischemic Preconditioning for Heart Surgery. *N Engl J Med.* 2015;373(15):1397–407. 10.1056/NEJMoa1413579 26436208

[46] Hausenloy DJ Candilio L Evans R: Remote Ischemic Preconditioning and Outcomes of Cardiac Surgery. *N Engl J Med.* 2015;373(15):1408–17. 10.1056/NEJMoa1413534 26436207

[47] Nashef S Roques F Hammill BG: Validation of European System for Cardiac Operative Risk Evaluation (EuroSCORE) in North American cardiac surgery. *Eur J Cardiothorac Surg.* 2002;22(1):101–5. 10.1016/s1010-7940(02)00208-7 12103381

[48] Nashef SAM Roques F Michel P: European system for cardiac operative risk evaluation (EuroSCORE). *Eur J Cardiothorac Surg.* 1999;16(1):9–13. 10.1016/s1010-7940(99)00134-7 10456395

[49] de Hert S Moerman A: Anesthetic Preconditioning: Have We Found the Holy Grail of Perioperative Cardioprotection? *J Cardiothorac Vasc Anesth.* 2018;32(3):1135–6. 10.1053/j.jvca.2018.01.001 29398375

[50] Raphael J Rivo J Gozal Y: Isoflurane-induced myocardial preconditioning is dependent on phosphatidylinositol-3-kinase/Akt signalling. *Br J Anaesth.* 2005;95(6):756–63. 10.1093/bja/aei264 16286350

[51] Deferrari G Bonanni A Bruschi M: Remote ischaemic preconditioning for renal and cardiac protection in adult patients undergoing cardiac surgery with cardiopulmonary bypass: Systematic review and meta-analysis of randomized controlled trials. *Nephrol Dial Transplant.* 2018;33(5):813–24. 10.1093/ndt/gfx210 28992285

[52] Gidday JM Fitzgibbons JC Shah AR: Neuroprotection from ischemic brain injury by hypoxic preconditioning in the neonatal rat. *Neurosci Lett.* 1994;168(1–2):221–4. 10.1016/0304-3940(94)90455-3 8028780

[53] Semenza GL: Pharmacologic Targeting of Hypoxia-Inducible Factors. *Annu Rev Pharmacol Toxicol.* 2019;59:379–403. 10.1146/annurev-pharmtox-010818-021637 30625281

[54] Wenger RH Stiehl DP Camenisch G: Integration of oxygen signaling at the consensus HRE. *Sci STKE.* 2005;2005(306):re12. 10.1126/stke.3062005re12 16234508

[55] Bishop T Ratcliffe PJ: HIF hydroxylase pathways in cardiovascular physiology and medicine. *Circ Res.* 2015;117(1):65–79. 10.1161/CIRCRESAHA.117.305109 26089364 PMC4501273

[56] Dhillon S: Roxadustat: First Global Approval. *Drugs.* 2019;79(5):563–72. 10.1007/s40265-019-01077-1 30805897

[57] Chen N Hao C Peng X: Roxadustat for Anemia in Patients with Kidney Disease Not Receiving Dialysis. *N Engl J Med.* 2019;381(11):1001–10. 10.1056/NEJMoa1813599 31340089

[58] Chen N Hao C Liu B-C: Roxadustat Treatment for Anemia in Patients Undergoing Long-Term Dialysis. *N Engl J Med.* 2019;381(11):1011–22. 10.1056/NEJMoa1901713 31340116

[59] Coyne DW Roger SD Shin SK: ANDES: A Phase 3, Randomized, Double-Blind, Placebo Controlled Study of the Efficacy and Safety of Roxadustat for the Treatment of Anemia in CKD Patients Not on Dialysis (Abstract at kidney week). *J Am Soc Nephrol.* 2019;30:822–823. Reference Source

[60] Charytan C Manllo-Karim R Martin ER: SIERRAS: A Phase 3, Open-Label, Randomized, Active-Controlled Study of the Efficacy and Safety of Roxadustat in the Maintenance Treatment of Anemia in Subjects with ESRD on Stable Dialysis (Abstract at kidney week). *J Am Soc Nephrol.* 2019;30:822 Reference Source

[61] Fishbane S El-Shahawy MA Pecoits-Filho R: OLYMPUS: A Phase 3, Randomized, Double-Blind, Placebo-Controlled, International Study of Roxadustat Efficacy in Patients with Non-Dialysis-Dependent (NDD) CKD and Anemia (Abtract at kidney week). *J Am Soc Nephro.* 2019;30:6.

[62] McCay CM Crowell MF Maynard LA: The Effect of Retarded Growth Upon the Length of Life Span and Upon the Ultimate Body Size: One Figure. *J Nutr.* 1935;10(1):63–79. 10.1093/jn/10.1.63 2520283

[63] Colman RJ Anderson RM Johnson SC: Caloric restriction delays disease onset and mortality in rhesus monkeys. *Science.* 2009;325(5937):201–4. 10.1126/science.1173635 19590001 PMC2812811

[64] Fontana L Partridge L: Promoting health and longevity through diet: from model organisms to humans. *Cell.* 2015;161(1):106–18. 10.1016/j.cell.2015.02.020 25815989 PMC4547605

[65] Kim CH Lee EK Choi YJ: Short-term calorie restriction ameliorates genomewide, age-related alterations in DNA methylation. *Aging Cell.* 2016;15(6):1074–81. 10.1111/acel.12513 27561685 PMC6398531

[66] Tiku V Jain C Raz Y: Small nucleoli are a cellular hallmark of longevity. *Nat Commun.* 2017;8:16083. 10.1038/ncomms16083 28853436 PMC5582349

[67] McKiernan SH Tuen VC Baldwin K: Adult-onset calorie restriction delays the accumulation of mitochondrial enzyme abnormalities in aging rat kidney tubular epithelial cells. *Am J Physiol Renal Physiol.* 2007;292(6):F1751–60. 10.1152/ajprenal.00307.2006 17344189

[68] Lee C Safdie FM Raffaghello L: Reduced levels of IGF-I mediate differential protection of normal and cancer cells in response to fasting and improve chemotherapeutic index. *Cancer Res.* 2010;70(4):1564–72. 10.1158/0008-5472.CAN-09-3228 20145127 PMC2836202

[69] Harputlugil E Hine C Vargas D: The TSC complex is required for the benefits of dietary protein restriction on stress resistance *in vivo*. *Cell Rep.* 2014;8(4):1160–70. 10.1016/j.celrep.2014.07.018 25131199 PMC4260622

[70] Yu D Tomasiewicz JL Yang SE: Calorie-Restriction-Induced Insulin Sensitivity Is Mediated by Adipose mTORC2 and Not Required for Lifespan Extension. *Cell Rep.* 2019;29(1):236–248.e3. 10.1016/j.celrep.2019.08.084 31577953 PMC6820997

[71] Arabi YM Aldawood AS Haddad SH: Permissive Underfeeding or Standard Enteral Feeding in Critically Ill Adults. *N Engl J Med.* 2015;372(25):2398–408. 10.1056/NEJMoa1502826 25992505

[72] Arabi Y Jawdat D Bouchama A: Permissive underfeeding, cytokine profiles and outcomes in critically ill patients. *PLoS One.* 2019;14(1):e0209669. 10.1371/journal.pone.0209669 30615631 PMC6322779

[73] Jongbloed F de Bruin RW Klaassen RA: Short-Term Preoperative Calorie and Protein Restriction Is Feasible in Healthy Kidney Donors and Morbidly Obese Patients Scheduled for Surgery. *Nutrients.* 2016;8(5):pii: E306. 10.3390/nu8050306 27213441 PMC4882718

[74] van Ginhoven TM de Bruin RW Timmermans M: Pre-operative dietary restriction is feasible in live-kidney donors. *Clin Transplant.* 2011;25(3):486–94. 10.1111/j.1399-0012.2010.01313.x 20718826

[75] Grundmann F Müller RU Reppenhorst A: Preoperative Short-Term Calorie Restriction for Prevention of Acute Kidney Injury After Cardiac Surgery: A Randomized, Controlled, Open-Label, Pilot Trial. *J Am Heart Assoc.* 2018;7(6): pii: e008181. 10.1161/JAHA.117.008181 29535139 PMC5907569

[76] Grundmann F Müller RU Hoyer-Allo KJR: Dietary restriction for prevention of contrast-induced acute kidney injury in patients undergoing percutaneous coronary angiography: a randomized controlled trial. *Sci Rep.* 2020;10: 5202. 10.1038/s41598-020-61895-2 PMC7089976 32251303

[77] Robertson LT Treviño-Villarreal JH Mejia P: Protein and Calorie Restriction Contribute Additively to Protection from Renal Ischemia Reperfusion Injury Partly via Leptin Reduction in Male Mice. *J Nutr.* 2015;145(8):1717–27. 10.3945/jn.114.199380 26041674 PMC4516761

[78] Guo F Cavener DR: The GCN2 eIF2alpha kinase regulates fatty-acid homeostasis in the liver during deprivation of an essential amino acid. *Cell Metab.* 2007;5(2):103–14. 10.1016/j.cmet.2007.01.001 17276353

[79] Kamata S Yamamoto J Kamijo K: Dietary deprivation of each essential amino acid induces differential systemic adaptive responses in mice. *Mol Nutr Food Res.* 2014;58(6):1309–21. 10.1002/mnfr.201300758 24668850

[80] Peng W Robertson L Gallinetti J: Surgical stress resistance induced by single amino acid deprivation requires *Gcn2* in mice. *Sci Transl Med.* 2012;4(118):118ra11–118ra11. 10.1126/scitranslmed.3002629 22277968 PMC3535286

[81] Hine C Harputlugil E Zhang Y: Endogenous hydrogen sulfide production is essential for dietary restriction benefits. *Cell.* 2015;160(1–2):132–44. 10.1016/j.cell.2014.11.048 25542313 PMC4297538

[82] Jongbloed F Saat TC Verweij M: A signature of renal stress resistance induced by short-term dietary restriction, fasting, and protein restriction. *Sci Rep.* 2017;7:40901. 10.1038/srep40901 28102354 PMC5244361

[83] Wang WJ Cai GY Ning YC: Hydrogen sulfide mediates the protection of dietary restriction against renal senescence in aged F344 rats. *Sci Rep.* 2016;6:30292. 10.1038/srep30292 27456368 PMC4960595

[84] Yang G Wu L Jiang B: H _2_S as a physiologic vasorelaxant: hypertension in mice with deletion of cystathionine gamma-lyase. *Science.* 2008;322(5901):587–90. 10.1126/science.1162667 18948540 PMC2749494

[85] Miller DL Roth MB: Hydrogen sulfide increases thermotolerance and lifespan in Caenorhabditis elegans. *Proc Natl Acad Sci U S A.* 2007;104(51):20618–22. 10.1073/pnas.0710191104 18077331 PMC2154480

[86] Sparatore A Santus G Giustarini D: Therapeutic potential of new hydrogen sulfide-releasing hybrids. *Expert Rev Clin Pharmacol.* 2014;4(1):109–21. 10.1586/ecp.10.122 22115352

[87] Orentreich N Matias JR DeFelice A: Low methionine ingestion by rats extends life span. *J Nutr.* 1993;123(2):269–74. 8429371 10.1093/jn/123.2.269

[88] Longo VD Mattson MP: Fasting: molecular mechanisms and clinical applications. *Cell Metab.* 2014;19(2):181–92. 10.1016/j.cmet.2013.12.008 24440038 PMC3946160

[89] Cheng CW Villani V Buono R: Fasting-Mimicking Diet Promotes Ngn3-Driven β-Cell Regeneration to Reverse Diabetes. *Cell.* 2017;168(5):775–788.e12. 10.1016/j.cell.2017.01.040 28235195 PMC5357144

[90] Wei M Brandhorst S Shelehchi M: Fasting-mimicking diet and markers/risk factors for aging, diabetes, cancer, and cardiovascular disease. *Sci Transl Med.* 2017;9(377): pii: eaai8700. 10.1126/scitranslmed.aai8700 28202779 PMC6816332

[91] Choi IY Piccio L Childress P: A Diet Mimicking Fasting Promotes Regeneration and Reduces Autoimmunity and Multiple Sclerosis Symptoms. *Cell Rep.* 2016;15(10):2136–46. 10.1016/j.celrep.2016.05.009 27239035 PMC4899145

[92] Stranahan AM Mattson MP: Recruiting adaptive cellular stress responses for successful brain ageing. *Nat Rev Neurosci.* 2012;13(3):209–16. 10.1038/nrn3151 22251954 PMC4084510

[93] Dong D Cai GY Ning YC: Alleviation of senescence and epithelial-mesenchymal transition in aging kidney by short-term caloric restriction and caloric restriction mimetics via modulation of AMPK/mTOR signaling. *Oncotarget.* 2017;8(10):16109–21. 10.18632/oncotarget.14884 28147330 PMC5369951

[94] Saxton RA Sabatini DM: mTOR Signaling in Growth, Metabolism, and Disease. *Cell.* 2017;168(6):960–76. 10.1016/j.cell.2017.02.004 28283069 PMC5394987

[95] Mizushima N Levine B Cuervo AM: Autophagy fights disease through cellular self-digestion. *Nature.* 2008;451(7182):1069–75. 10.1038/nature06639 18305538 PMC2670399

[96] Brown-Borg HM Buffenstein R: Cutting back on the essentials: Can manipulating intake of specific amino acids modulate health and lifespan? *Ageing Res Rev.* 2017;39:87–95. 10.1016/j.arr.2016.08.007 27570078 PMC5571732

[97] Fontana L Cummings NE Arriola Apelo SI: Decreased Consumption of Branched-Chain Amino Acids Improves Metabolic Health. *Cell Rep.* 2016;16(2):520–30. 10.1016/j.celrep.2016.05.092 27346343 PMC4947548

[98] Solon-Biet SM McMahon AC Ballard JW: The ratio of macronutrients, not caloric intake, dictates cardiometabolic health, aging, and longevity in ad libitum-fed mice. *Cell Metab.* 2014;19(3):418–30. 10.1016/j.cmet.2014.02.009 24606899 PMC5087279

[99] Solon-Biet SM Cogger VC Pulpitel T: Branched chain amino acids impact health and lifespan indirectly via amino acid balance and appetite control. *Nat Metab.* 2019;1(5):532–45. 10.1038/s42255-019-0059-2 31656947 PMC6814438

[100] Wang TJ Larson MG Vasan RS: Metabolite profiles and the risk of developing diabetes. *Nat Med.* 2011;17(4):448–53. 10.1038/nm.2307 21423183 PMC3126616

[101] Juricic P Grönke S Partridge L: Branched-Chain Amino Acids Have Equivalent Effects to Other Essential Amino Acids on Lifespan and Aging-Related Traits in *Drosophila*. *J Gerontol A Biol Sci Med Sci.* 2020;75(1):24–31. 10.1093/gerona/glz080 30891588 PMC6909895

[102] Solon-Biet SM Walters KA Simanainen UK: Macronutrient balance, reproductive function, and lifespan in aging mice. *Proc Natl Acad Sci U S A.* 2015;112(11):3481–6. 10.1073/pnas.1422041112 25733862 PMC4371964

[103] Lee SJ Murphy CT Kenyon C: Glucose shortens the life span of *C. elegans* by downregulating DAF-16/FOXO activity and aquaporin gene expression. *Cell Metab.* 2009;10(5):379–91. 10.1016/j.cmet.2009.10.003 19883616 PMC2887095

[104] Verweij M van de Ven M Mitchell JR: Glucose supplementation does not interfere with fasting-induced protection against renal ischemia/reperfusion injury in mice. *Transplantation.* 2011;92(7):752–8. 10.1097/TP.0b013e31822c6ed7 21926943

[105] Roberts MN Wallace MA Tomilov AA: A Ketogenic Diet Extends Longevity and Healthspan in Adult Mice. *Cell Metab.* 2017;26(3):539–546.e5. 10.1016/j.cmet.2017.08.005 28877457 PMC5609489

[106] Shimazu T Hirschey MD Newman J: Suppression of oxidative stress by β-hydroxybutyrate, an endogenous histone deacetylase inhibitor. *Science.* 2013;339(6116):211–4. 10.1126/science.1227166 23223453 PMC3735349

[107] Neal EG Chaffe H Schwartz RH: The ketogenic diet for the treatment of childhood epilepsy: A randomised controlled trial. *Lancet Neurol.* 2008;7(6):500–6. 10.1016/S1474-4422(08)70092-9 18456557

[108] Torres JA Kruger SL Broderick C: Ketosis Ameliorates Renal Cyst Growth in Polycystic Kidney Disease. *Cell Metab.* 2019;30(6):1007–1023.e5. 10.1016/j.cmet.2019.09.012 31631001 PMC6904245

[109] Kim JY Park JH Kim K: Pharmacological Inhibition of Caspase-1 Ameliorates Cisplatin-Induced Nephrotoxicity through Suppression of Apoptosis, Oxidative Stress, and Inflammation in Mice. *Mediators Inflamm.* 2018;2018:6571676. 10.1155/2018/6571676 30670928 PMC6323438

[110] Huang S You J Wang K: *N*-Acetylcysteine Attenuates Cisplatin-Induced Acute Kidney Injury by Inhibiting the C5a Receptor. *Biomed Res Int.* 2019;2019:4805853. 10.1155/2019/4805853 31111056 PMC6487137

[111] Späth MR Hoyer-Allo KJR Bohl K: Deciphering the Molecular Mechanisms Underlying Nephroprotection by Hypoxia-Signalling: A Comparative Analysis of Prolyl Hydroxylase Inhibition and Hypoxic Preconditioning (Abstract at kidney week). *J Am Soc Nephrol.* 2019;30:786.

[112] Johnsen M Kubacki T Yeroslaviz A: The Integrated RNA Landscape of Renal Preconditioning against Ischemia-Reperfusion Injury. *J Am Soc Nephrol.* 2020.pii: ASN.2019050534. 10.1681/ASN.2019050534 32111728 PMC7191926

